# Influence of Immune Microenvironment on Diagnosis and Prognosis of Head and Neck Squamous Cell Carcinoma

**DOI:** 10.3389/fonc.2021.604784

**Published:** 2021-03-17

**Authors:** Guohong Liu, Chunjue Yuan, Jiaojiao Ma, Yunbao Pan, Haibo Xu

**Affiliations:** ^1^ Department of Radiology, Zhongnan Hospital of Wuhan University, Wuhan University, Wuhan, China; ^2^ Department of Laboratory Medicine, Zhongnan Hospital of Wuhan University, Wuhan University, Wuhan, China; ^3^ Center for Gene Diagnosis, Zhongnan Hospital of Wuhan University, Wuhan University, Wuhan, China

**Keywords:** head and neck squamous cell carcinoma, tumor microenvironment, immune scores, prognosis, TCGA database

## Abstract

Head and neck squamous cell carcinoma (HNSCC) is an immunosuppressive malignancy accompanied by noted alterations in various immune cells and cytokines. Recognition of the immune system’s role in contributing to cancer development is an important advancement in our original understanding of carcinoma. We obtained HNSCC gene expression and clinical data from The Cancer Genome Atlas (TCGA) database. We assessed the relative proportion of 22 Infiltrating immune cell types in both HNSCC and adjacent non-cancer tissues using Cell-type Identification By Estimating Relative Subsets Of RNA Transcripts (CIBERSORT) method, identifying the influence of the immune cells content in tumor staging and survival prediction. We further predicted the tumor purity, and the presence of infiltrating stromal/immune cells in HNSCC tissues using Estimation of STromal and Immune cells in Malignant Tumor tissues using Expression data (ESTIMATE) algorithm, identifying its potential correlation with patient survival. Stromal and immune score-associated differentially expressed genes (DEGs) were subsequently verified and their roles in immune response were displayed by functional enrichment analysis and protein-protein interaction (PPI) network. Our research demonstrated the underlying association between the immune microenvironment and HNSCC, and the results were intended to serve as valuable terms for HNSCC diagnosis, prognosis, and targeted immune therapy.

## Introduction

Head and neck squamous cell carcinoma (HNSCC), accounting for nearly 95% of head and neck cancer cases, is the sixth most common type of malignant tumor worldwide. About 635,000 cases are newly diagnosed every year with Chinese patients’ occupancy at more than 12% (1), and only 50–60% of patients are alive at 5 years after diagnosis (2). Tumor cells are first recognized as foreign invaders by the host immune cells and then are effectively destroyed by an activated immune system (3). Several immune system alterations occur in HNSCC patients, suggesting that this cancer is an immunosuppressive malignancy (3, 4), the adaptive immune response is suppressed in HNSCC through overexpression of cytokines, triggering apoptosis of T cells, and alterations in antigen processing machinery (5, 6). A better understanding of the immune microenvironment in cancer can promote immunotherapy for the treatment of carcinoma (7).

Tumor microenvironment of cancer patients is composed of cancer cells, adjacent epithelial, stromal, and immune cells (8). The cellular elements of tumor microenvironment often coevolve with tumor suppression or progression. Derangements in various immune cells contribute to cancer suppression or progression eventually. HNSCC is among the most immune infiltrated tumors as reported previously (9). Anthony R. Cillo et al. had assessed the transcriptional profiles and identified immune cell clusters within tumors of HNSCC on single cell level (10). In this study, we first assessed the relative proportion of 22 Infiltrating immune cell types in both HNSCC and adjacent non-cancer tissues from TCGA database using Cell-type Identification By Estimating Relative Subsets Of RNA Transcripts (CIBERSORT) method, identifying the influence of the immune cells content in tumor staging and survival prediction. It provides valuable targets for tumor immune therapy. Besides, an algorithm called ESTIMATE (Estimation of STromal and Immune cells in MAlignant Tumor tissues using Expression data) can be applied to tumor purity prediction based on single sample gene set enrichment analysis which generates three scores: stromal score that captures the presence of stroma in tumor tissue, immune score that represents the infiltration of immune cells in tumor tissue, and ESTIMATE score that infers tumor purity (11). Increased application of the algorithm in multiple types of research provided us a new tool for diagnosis and prognosis of cancer (12, 13). Bin Liang et al. also conducted the immune cell type identification of HNSCC patient samples by CIBERSORT. They further analyzed differentially expressed genes (DEGs) by grouping samples into HNSCC tissues and adjacent non-cancer tissues, and conducted enrichment analysis based on those DEGs (14). However, the DEGs generated by this kind of grouping can be hardly related to immune cell type and tumor immune microenvironment. To distinguish useful genes that are differentially expressed in HNSCC specific immune microenvironment which may influence tumor immune response, we assessed stromal, immune, and ESTIMATE score of HNSCC samples from TCGA database using ESTIMATE algorithm. Stromal and immune score-associated DEGs were subsequently verified by dividing the samples into high and low stromal/immune score groups with median score as the cutoff. The role of these DEGs in immune response were subsequently displayed by protein-protein interaction (PPI) network, as well as GO and KEGG functional enrichment analysis. Our research demonstrated the underlying association between immune microenvironment and HNSCC, and the results were intended to serve as valuable terms in HNSCC diagnosis, prognosis and targeted immune therapy.

## Materials and Methods

### Database

We obtained all the gene expression and clinical data of HNSCC patients from TCGA data portal (https://tcga-data.nci.nih.gov/tcga/). There are 501 HNSCC patients in total, with RNA expression data from 502 HNSCC cancer tissues and 44 adjacent non-cancer tissues. All of the 501 patients had complete survival data, while only 433 patients had tumor stage information.

### Immune Infiltration Analysis

We used CIBERSORT (Cell-type Identification By Estimating Relative Subsets Of RNA Transcripts) method to characterize the infiltrating immune cell types from complex tissues based on RNA sequencing data. A validated leukocyte gene signature matrix (LM22) was used to identify the 22 functionally defined immune cell subtypes. The filter criteria of each sample is set as the CIBERSORT calculation of P < 0.05, indicating that the inferred proportion of each infiltrating immune cell subtype is fairly accurate and suitable for further analysis. We used R packages to visualize the 22 immune cells content in both HNSCC samples and adjacent non-cancer samples, and illustrated the relationship of immune cells content with patient stage.

### Tumor Purity Analysis

To evaluate tumor purity, and the presence of infiltrating stromal and immune cells in HNSCC tissues, we calculated stromal score, immune score, and ESTIMATE score by applying the ESTIMATE (Estimation of STromal and Immune cells in Malignant Tumor tissues using Expression data) algorithm to the downloaded gene expression data of 502 HNSCC samples. Stromal score captures the presence of stroma in tumor tissue, immune score represents the infiltration of immune cells in tumor tissue, and ESTIMATE score infers tumor purity (11).

### Identification of Differentially Expressed Genes (DEGs)

We analyzed all the data with the help of R programming language, and “limma” package in R was applied to study the DEGs between HNSCC samples with high stromal/immune score (n = 251) and low stromal/immune score (n = 251) groups by setting logFC > 1 and FDR < 0.05. Commonly upregulated or downregulated intersect genes of high stromal score and immune score groups were extracted using R package of “VennDiagram”.

### Overall Survival Analysis

We applied Kaplan-Meier plots using R package of “survival” to show the relationship between patients’ overall survival and different immune cells content, Stromal/Immune/ESTIMATE scores, or gene expression levels of DEGs. The results were tested by the log-rank test.

### Construction of PPI Network

We constructed the PPI network of the differentially expressed intersect genes in the high stromal score and immune score groups with the highest confidence of 0.9 on the online STRING database (https://string-db.org/), a database of known and predicted protein-protein interactions. The interactions include direct (physical) and indirect (functional) associations; they stem from computational prediction, from knowledge transfer between organisms, and interactions aggregated from other (primary) databases. We further filtered out the top 30 critical genes with 16 or more connecting nodes in the PPI network using Cytoscape software.

### Enrichment Analysis of DEGs

Functional enrichment analysis of Gene Ontology (GO) and Kyoto Encyclopedia of Genes and Genomes (KEGG) were performed by using R packages of “clusterProfiler” and “enrichplot”. False discovery rate (FDR) < 0.05 was considered as significant enrichment.

### Statistical Analysis

All the statistical analyses in this study are performed using R v3.6.2 and publicly available Bioconductor R packages (https://www.bioconductor.org/). Student’s t-test and one-way analysis of variance (ANOVA) were utilized to compare continuous (different proportions of 22 immune cell types between HNSC tissues and adjacent non-cancer tissues) and discrete (clinical staging characteristics in HNSC patients) variables, respectively. Overall survival curves were calculated by Kaplan–Meier method and tested by log-rank test. In all appropriate instances, p values were corrected for multiple comparisons using a false discovery rate with a threshold of <0.05 for statistical significance.

## Result

### Infiltrating Immune Cell Types Associated With Clinical Stage of HNSCC Cases

**Figure 1:**
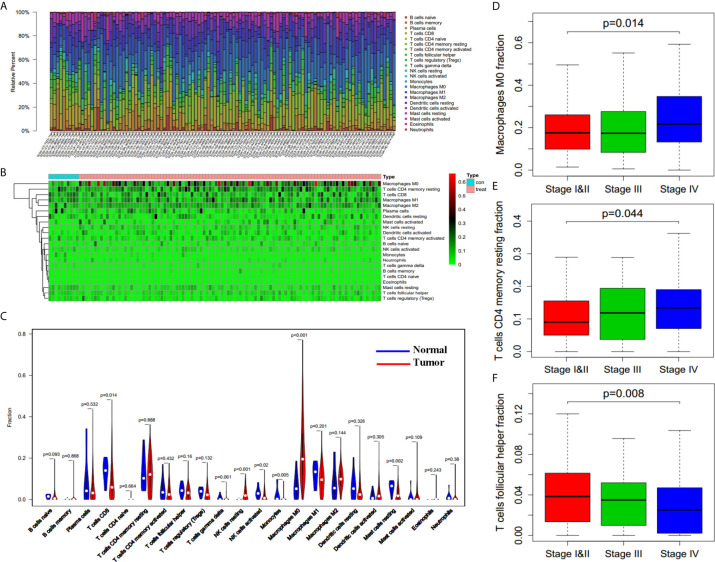
Filtrating immune cell types associated with HNSCC. **(A)** Variation of different immune cells in content under normal and pathological state derived from TCGA database (n = 110 for presentation from 430 samples). **(B)** Heatmap of different immune cells content in control group (normal tissues, n = 10) and tumor group (tumor tissues, n = 100 for presentation from 420 tumor samples). **(C)** Differences between control group (blue, normal tissues, n = 10) and tumor group (red, tumor tissues, n = 420) in immune cells content. **(D–F)** M0 Macrophages fraction **(D)**, resting CD4 memory T cells fraction **(E)**, and follicular helper T cells fraction **(F)** in patients with different clinical stages (n = 362, p = 0.014, p = 0.044, p = 0.008).

RNA expression data from 502 HNSCC cancer tissues and 44 adjacent non-cancer tissues were obtained from the TCGA database. Clinical information of 501 HNSCC patients is also derived. Among all the HNSCC patients, 133 cases were female and 368 patients were male. These HNSCC patients were divided into three clinical stages according to their pathological condition, including 96 cases of stage I and II, 78 cases of stage III, and 259 cases of stage IV (with 68 cases of unknown stage). By applying the CIBERSORT method, we have learned the complicated component of infiltrating immune cells in the samples (**Figure 1A**). Ten adjacent non-cancer tissues (normal samples) and 420 tumor tissues (tumor samples) were filtered out which can be evaluated fairly accurately by CIBERSORT method with P < 0.05. We quantified the amount of different immune cells in both groups, trying to discover the immune cell types associated with carcinoma (**Figures 1B, C**). It was found that resting NK cells and M0 macrophages were significantly increased in HNSCC, while CD8 T cells, activated NK cells, and resting mast cells were significantly decreased in HNSCC with P < 0.05 (**Figure 1C**). These data indicated that the aberrantly expressed immune cells may contribute to the tumorigenesis and tumor progression of HNSCC.

Subsequently, we assessed whether there was an underlying correlation between infiltrating immune cell types and clinical stages (362 patients with stage information). We compared the proportion of immune cells in patients with different clinical stages, and found that the proportion of both M0 macrophages (**Figure 1D**, p = 0.014) and resting CD4 memory T cells (**Figure 1E**, p = 0.044) are increased in higher stage, whereas the proportion of follicular helper T cells (**Figure 1F**, p = 0.008) is decreased in higher stage. The results indicate that infiltrating immune cells influenced the process of cancer aggravation.

### Tumor Microenvironment Is Significantly Associated With Overall Survival of HNSCC Patients

**Figure 2:**
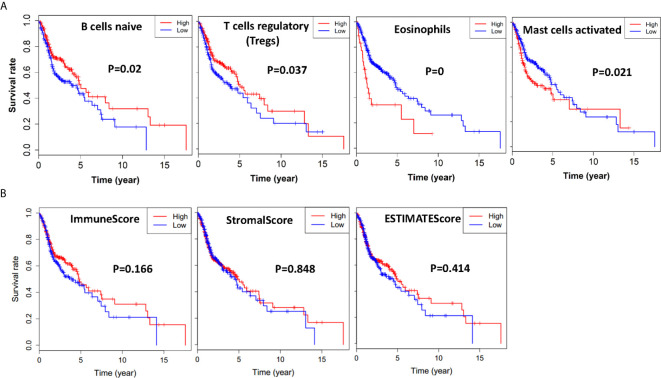
Immune cells, immune scores, stromal scores, and ESTIMATE scores are associated with HNSCC overall survival. **(A)** HNSCC cases were divided into two groups based on their naïve B cells, Tregs, eosinophils, or activated mast cells content: the top half of 210 cases had more immune cells and the bottom half of 210 cases had less immune cells. As is shown in the Kaplan-Meier survival curves, the median survival of the high content group which had more naïve B cells and Treg cells is significantly longer than the low content group with p = 0.02 and p = 0.037, respectively. On the contrary, the median survival of the high content group which had more eosinophils, and mast cells is shorter than the low content group with p = 0.00 and p = 0.021, respectively. **(B)** HNSCC cases were divided into two groups based on their immune scores, stromal scores, or ESTIMATE score: the top half of 251 cases with higher scores and the bottom half of 250 cases with lower scores. As is shown in the Kaplan-Meier survival curves, the median survival of the high score group is relatively longer than the low score group (p = 0.166, p = 0.848, p = 0.418).

To find out whether there was a correlation between tumor microenvironment and clinical overall survival, we divided all the HNSCC cases into halves according to every immune cell content with median value as the cutoff, and compared the length of survival years between these two groups. Immune cell types that induced notable survival differences were thus demonstrated. As shown in Kaplan-Meier survival curves (**Figure 2A**), patients who had more naïve B cells (p = 0.02) and regulatory T cells (Tregs) lived longer (p = 0.037), whereas more eosinophils (p = 0.00) and activated mast cells (p = 0.021) shortened their lifespan.

Based on ESTIMATE algorithm, we further divided all the HNSCC cases into halves by their Stromal score, Immune score, and ESTIMATE score, with median value as the cutoff. As shown in **Figure 2B**, the longer median overall survival was positively linked with a higher immune score (p = 0.166). The result was consistent with the stromal score group that median overall survival of cases with low score group was shorter (p = 0.848), and so was the ESTIMATE score groups (p = 0.418), although statistically not significant.

### Comparison of Gene Expression Profile With Different Stromal Score and Immune Score in HNSCC

**Figure 3:**
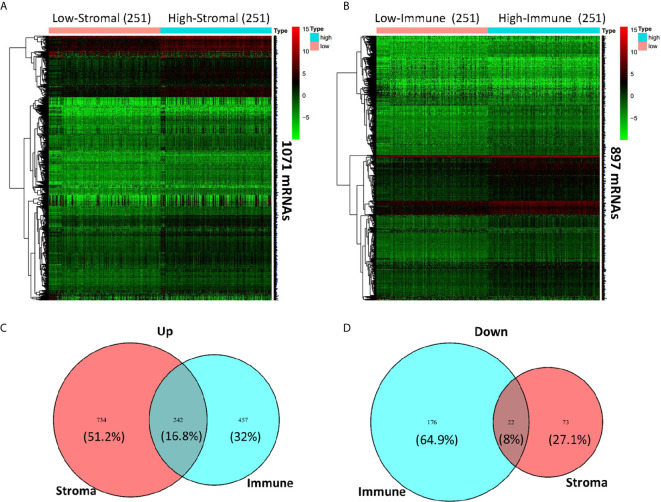
Comparison of gene expression profile with immune scores and stromal scores in HNSCC. Heatmaps were drawn based on the average linkage method and Pearson distance measurement method. Genes with higher expression are shown in red and lower expression is shown in green. **(A)** Heatmap of the DEGs of stromal scores of top half (high score, n = 251) *vs.* bottom half (low score, n = 251). A total of 1,071 DEGs were derived with logFC > 1 and FDR < 0.05. **(B)** Heatmap of the DEGs of immune scores of top half (high score, n = 251) *vs.* bottom half (low score, n = 251). A total of 897 DEGs were derived with logFC > 1 and FDR < 0.05. **(C, D)** Venn diagrams showing the intersect 242 upregulated **(C)** or 22 downregulated **(D)** DEGs in both stromal and immune score groups.

To figure out the corresponding relationship between global gene expression profiles and assess scores based on ESTIMATE, we compared all the HNSCC cases from TCGA database by stromal score and immune score, and drew gene expression heatmaps (**Figures 3A, B**). Gene expression differences can be observed visually and distinctly between high (n = 251) and low (n = 251) stromal score and immune score groups. A total of 1,071 and 897 significant differentially expressed mRNAs were filtered out in stromal score and immune score groups, respectively, with logFC > 1 and FDR < 0.05. The heatmap grouped by Immune score showed that 699 genes were upregulated, whereas another 198 genes were downregulated in the high immune score group as compared to the low Immune score group (**Figures 3B–D**). As for the stromal score group, 976 genes were upregulated and 95 genes were downregulated in the high stromal score group as compared to the low stromal score group (**Figures 3A, C, D**). As was shown in the Venn diagrams (**Figures 3C, D**), there were 242 intersect genes upregulated, and 22 intersect genes downregulated in the high stromal and immune score groups as compared to the corresponding low score groups. We also noticed that more than half of downregulated DEGs existed in the immune score group while the upregulated DEGs in the stromal score group account for 51.2%.

### Correlation of Expression of Individual DEGs in Overall Survival

**Figure 4:**
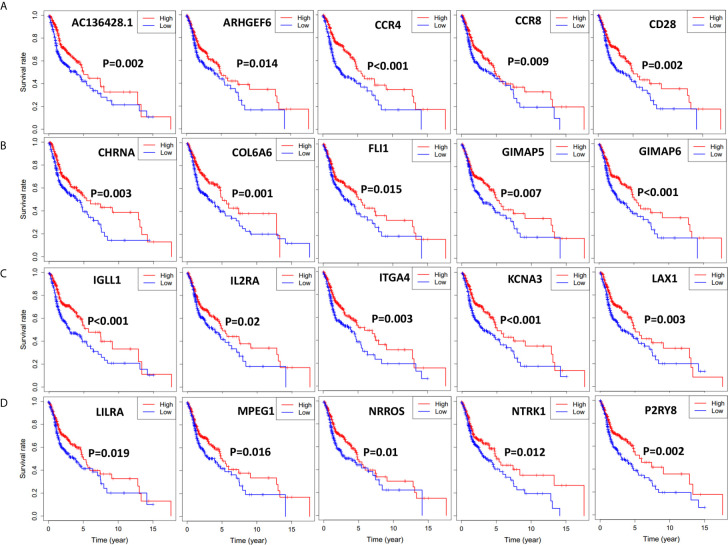
Significant DEGs associated with overall survival of 501 HNSCC patients, genes were listed from left to right. Single mRNA Survival prediction by the expression level of 20 Significant DEGs filtered out from the intersect genes in the Venn graph which showed significant differential survival rate with p value <0.02. The cutoffs of the high and low gene expression groups were the median value. **(A)** AC136428.1, ARHGEF6, CCR4, CCR8, CD28. **(B)** CHRNA, COL6A6, FLI1, GIMAP5, GIMAP6. **(C)** IGLL1, IL2RA, ITGA4, KCNA3, LAX1. **(D)** LILRA, MPEG1, NRROS, NTRK1, P2RY8.

**Figure 5:**
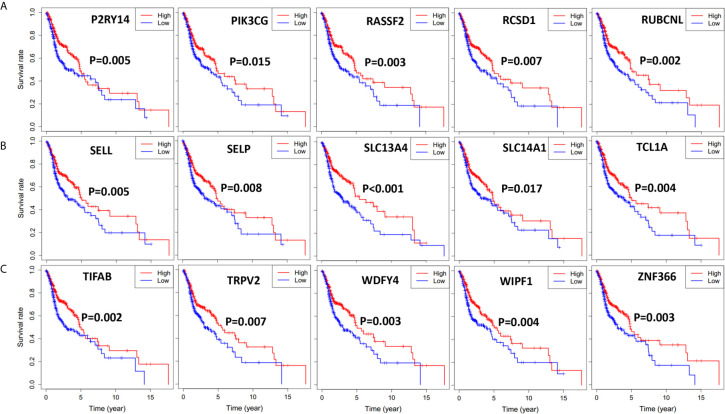
Significant DEGs associated with overall survival of 501 HNSCC patients, genes were listed from left to right. Single mRNA Survival prediction by the expression level of 15 Significant DEGs filtered out from the intersect genes in the Venn graph which showed significant differential survival rate with p value <0.02. The cutoffs of high and low gene expression groups were the median value. **(A)** P2RY14, PIK3CG, RASSF2, RCSD1, RUBCNL. **(B)** SELL, SELP, SLC13A4, SLC14A1, TCL1A. **(C)** TIFAB, TRPV2, WDFY4, WIPF1, ZNF366.

To find out the possible association between the 264 individual intersect DEGs and overall survival, we drew Kaplan-Meier survival curves according to the gene expression level with median expression value as the cutoff. Furthermore, genes with statistical significance (p < 0.02) were shown in **Figures 4** and **5**, which indicated that higher expression of those genes were correlated positively with longer survival.

### Protein-Protein Interactions Among Genes With Prognostic Value

**Figure 6:**
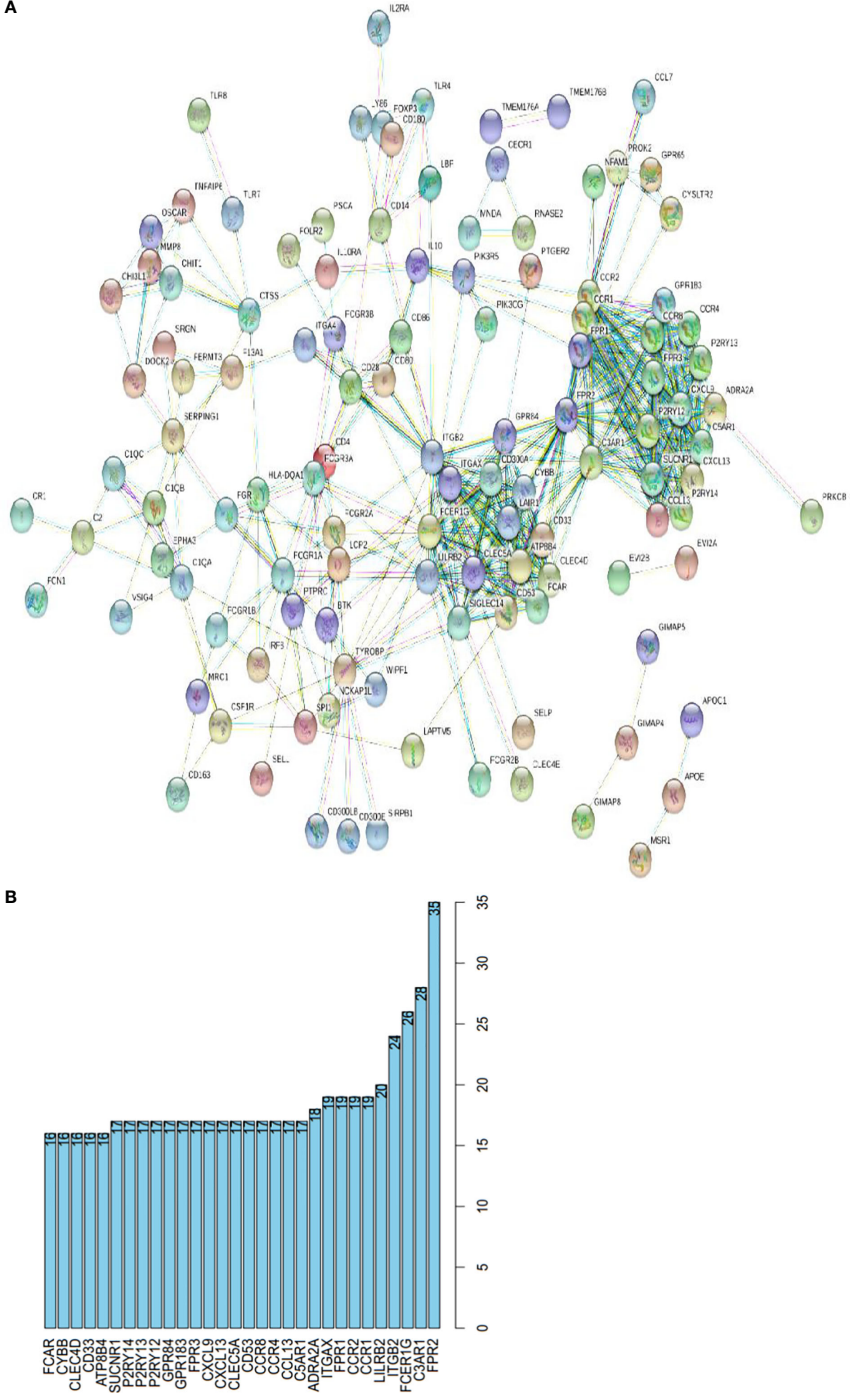
Protein-protein interactions among genes with prognostic value. **(A)** PPI network with the confidence of 0.9. **(B)** Top 30 genes involved in PPI network with more than 16 connecting nodes.

To further clarify the DEGs involved in the regulation network of immune responses, we obtained protein-protein interaction (PPI) networks using the online STRING database (**Figure 6A**). Top 30 remarkable genes with 16 or more connecting nodes in the network were filtered out using Cytoscape software and were shown in **Figure 6B**. Genes of FPR2, C3AR1, FCER1G, and ITGB2 had the highest degree values in the connection network.

### Functional Enrichment Analysis of Immune Related Genes With Prognostic Value

**Figure 7:**
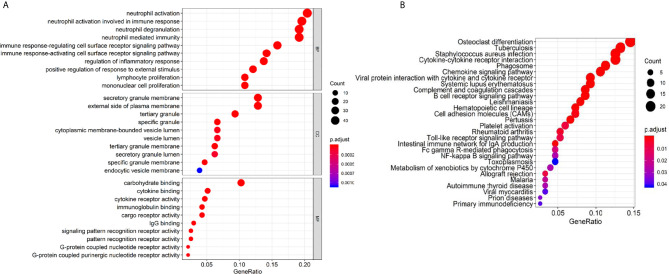
GO terms and KEGG pathway analysis of prognostic genes. **(A)** Biological process (BP), cellular component (CC), and molecular function (MF). **(B)** KEGG pathway.

Then we investigated gene annotation information of these DEGs by conducting Gene Ontology (GO) analysis (**Figure 7A**), which includes cellular component (CC), biological process (BP), and molecular function (MF). Among these terms, neutrophil activation, neutrophil degranulation, and neutrophil-mediated immunity are of great importance. Besides, we conducted Kyoto Encyclopedia of Genes and Genomes (KEGG) analysis and found 28 KEGG pathways involved in HNSCC process which showed significant enrichment with p value <0.05 (**Figure 7B**), including osteoclast differentiation, tuberculosis, *Staphylococcus aureus* infection, and immune-related pathways such as cytokine-cytokine receptor interaction, phagosome, chemokines signaling pathway, and B cell receptor signaling pathway.

## Discussion

Tumors reprogram their surroundings, creating a metabolically fertile environment to meet their high energy and anabolic requirements, and this process was aptly described by Paget as the “seed and soil” hypothesis (15). Through the tumor’s influence on the microenvironment, the immune system can be exploited to promote metastasis, angiogenesis, and growth of carcinoma. This complex milieu between tumor cells and cells surrounding the tumor provides necessary space and elements for tumor progression, and creates an immunosuppressive environment (16–18). It consists of the extracellular matrix, soluble molecules, and tumor stromal cells and keeps a dynamic balance all the time (19). Both immune cells and non-immune cells are likely to have an essential impact on the initiation, promotion, and progression of tumors that arise in the microenvironment (20).

In our study, we planned to distinguish related infiltrating non-tumor cells and genes which contributed to HNSCC development based on gene expression and clinical data of HNSCC patients on TCGA database. Firstly, we analyzed the content of infiltrating immune cells in 420 tumor tissues and 10 normal tissues by CIBERSORT, discovering the notable differences between them. Among the immune cells, resting NK cells and M0 macrophage were upregulated in tumor microenvironment, whereas CD8 T cells, activated NK cells, and resting mast cells were downregulated significantly with P < 0.05. We also found that M0 macrophages and resting CD4 memory T cells were positively associated with clinical stage, while follicular helper T cells negatively correlated with clinical stage. This is consistent with previous studies (21). What we should pay attention to is that M0 macrophages were statistically different in both preliminary diagnostic tests and late clinical staging, which indicates that they might be a vital immune cell to the diagnosis, prognosis, and targeted immune therapy of HNSCC.

The distribution, density, and functional status of the immune cells in tumor tissues and other comprehensive immunological factors can be independent prognostic factors of many cancers, including breast cancer (22), ovarian cancer (23), prostate cancer (24), and colorectal cancer (25). As cellular immunity is the main form of anti-tumor immunity, immune cells that exist in the tumor microenvironment play a significant role in anti-tumor immune response, and the activated immune system may be an effective treatment for cancer patients. T cells, natural killer cells (NK cells), and macrophages are the most critical effectors. Tumor-associated macrophages have long been identified as a potential inhibitor of tumor expansion, which play critical roles in anti-tumor immunity. They can clear tumor cells by killing them directly or activating the immune response of the body through presenting tumor-related antigen. Our study also found that the content of M0 macrophages is increased significantly in HNSCC tissue. It provided us a theoretical basis for exploring immune therapy strategy and finding innovative immunotherapy target for improving the survival of HNSCC patients.

Next, we performed Kaplan-Meier survival analysis to find out the relationship of survival with immune cells content. We calculated the stromal/immune score of HNSCC samples using ESTIMATE algorithm, classified the HNSCC samples into high and low stromal/immune score groups, and analyzed their association with clinical survival. We further performed gene differential expression analysis between high and low stromal/immune score groups, and filtered out the commonly upregulated or downregulated intersect genes for high stromal score and immune score groups as compared to low score groups, which may influence the tumor immune microenvironment and eventually patient diagnosis and prognosis. Among those intersect genes, we found 35 genes that influence patient survival significantly with p < 0.02, as shown in **Figures 4** and **5**. We further present the interplay of essential regulatory modules by constructing the PPI network among those intersect DEGs on an online STRING database. Among the top 30 genes with more than 16 connecting nodes, FPR2, ITGB2, and C3AR1 had the highest connecting nodes. CCR4, CCR8, ITGA4, and P2RY14 were upregulated in the high stromal/immune score group and the high level of their expression showed a significant correlation with longer patient survival with p < 0.01 as displayed in **Figures 4** and **5**. The formyl peptide receptor (FPR) family is highly expressed on the surface of some malignant tumor cells and is closely related to the occurrence, development, and metastasis of these tumors. FPR2, one of the famous receptors, has been reported to be involved in many kinds of carcinoma, including gastric cancer (26) and colorectal cancer (27). In HNSCC, FPR2 takes effect after the activation of annexin 1 (AnxAl) which is associated with metastasis of some invasive malignancies (28). It was reported that ITGB2, which is mainly expressed on the surface of leukocytes, can recruit tumor cells by combining ICAM (29). Complement receptor C3AR1 was also proved to promote an immunosuppressive microenvironment by limiting expansion and differentiation of alloreactive CD8+ T cell immunity (30). The role of FCER1G in HNSCC, one of the hub node genes, remains to be clarified. Overexpression of integrin α4 (ITGA4) has been reported in several cancers which mediates migration of cancerous cells (31, 32). Thus, inhibition of ITGA4 could be a therapeutic strategy.

Enriched KEGG pathways, such as cytokine-cytokine receptor interaction (33, 34), chemokines signaling pathway (35, 36), and B cell receptor signaling pathway, are closely related to the immune microenvironment in tumors and are critical for tumor immune responses. A complex network of chemokines and their receptors influences the development of primary tumors and metastases (37). It has been shown that Tregs strongly express CCR4, a chemokine receptor for CCL17 or CCL22, on their surface as compared to effector T cells in leukemia studies (38). CCL17 and CCL22 played an essential part in immune inhibition by gathering CD4^+^CD25^+^Tregs. This process has been identified to promote invasion and metastasis of some solid tumors such as stomach cancer and esophageal cancer (35, 36). Suppressive activity of regulatory T cells and upregulation of the CCR4 were reported to be observed in the peripheral blood sample of HNSCC patients (39–41). Our study showed that low level of Tregs and CCR4 were related to a worse prognosis of HNSCC patients, which is different from the above results.

Remarkably Enriched GO terms of neutrophil activation, neutrophil degranulation, and neutrophil-mediated immunity, all indicated that the immune microenvironment regulates HNSCC and are valuable for tumor immunology. It revealed the close relationship between stromal/immune related DEGs and tumor immune response. Human neutrophils contain a releasable membrane-bound organelle named the secretory vesicle and are endowed with three major types of cytoplasmic granules that modulate cell function, namely primary or azurophilic granules, secondary or specific granules, and tertiary or gelatinase granules (42). Mature neutrophils (polymorphonuclear neutrophils, PMNs; or neutrophil polymorphonuclear granulocytes) account for ~50–70% of all leukocytes in adult human peripheral blood, and are abundant in tumor microenvironments (43). Neutrophils constitute the first line of defense against invading pathogens and are the first leukocytes to migrate to sites of inflammation (44). The highly enriched biological process and cellular component of GO terms indicate a strong correlation of DEGs with immune responses.

In conclusion, we extracted the 22 immune cells content and gene expression of clinical HNSCC patients on TCGA database which is related to tumor microenvironment. We conducted the immune infiltrating analysis, tumor purity analysis, survival analysis, gene differential expression analysis, protein-protein-interaction analysis, and functional enrichment analysis of HNSCC, and analyzed the influence of immune microenvironment on stage and survival of HNSCC patients. The results can serve as valuable terms for HNSCC diagnosis, prognosis, and targeted immune therapy. Further exploiting individual genes or their combined effects on carcinoma can lead to novel insights into the relationship between tumor microenvironment and HNSCC diagnosis and prognosis.

## Data Availability Statement

The original contributions presented in the study are included in the article. Further inquiries can be directed to the corresponding authors.

## Author Contributions

Conceptualization: YP and HX. Methodology: YP and GL. Formal analysis: YP and GL. Investigation: GL, CY, YP, JM, and HX. Writing—original draft: GL and CY. Writing—review and editing: YP and GL. Supervision: YP and HX. All authors contributed to the article and approved the submitted version.

## Funding

This work was supported by the National Key R&D Program of China (2017YFC0108800), the National Natural Science Foundation of China (81872200, 31900558), the Natural Science Foundation of Hubei Province (2020CFB298, 2018CFB510), the Zhongnan Hospital of Wuhan University Science, Technology and Innovation Seed Fund (ZNPY2018090, ZNPY2019002), and the Fundamental Research Funds for the Central Universities (2042019kf0139, 2042018kf0091).

## Conflict of Interest

The authors declare that the research was conducted in the absence of any commercial or financial relationships that could be construed as a potential conflict of interest.
